# A Comparison of Podoplanin Expression in Oral Leukoplakia and Oral Squamous Cell Carcinoma: An Immunohistochemical Study

**DOI:** 10.7759/cureus.38467

**Published:** 2023-05-02

**Authors:** Vaishnavi Srinivasan, NDVN Shyam, G Kiran Kumar, Vaishali Narayen, Paremala Konda, Korra Swetha Rani

**Affiliations:** 1 Department of Oral Pathology, Government Dental College and Hospital, Hyderabad, IND

**Keywords:** podoplanin, oral squamous cell carcinoma, oral potentially malignant disorders, oral leukoplakia, immunohistochemistry

## Abstract

Introduction: Oral squamous cell carcinoma (OSCC) accounts for about 90% to 95% of all malignancies of the oral cavity.The majority of OSCCs are preceded by oral potentially malignant disorders (OPMDs). Podoplanin (PDPN) is a mucin-like small transmembrane glycoprotein. Alterations in PDPN immunoexpression have been reported in OPMDs and OSCCs.

Objective: The objectives of this study were to evaluate the role of PDPN immunoexpression in oral leukoplakia (OL) and different histological grades of OSCC and to assess the role of PDPN as a potential biomarker for predicting the risk of malignant transformation.

Materials and methodology: Immunohistochemical analysis for PDPN was performed in 45 histologically confirmed cases of formalin-fixed, paraffin-embedded specimens of different grades of OSCCs and 15 cases of OLs with 15 cases of the normal oral mucosa (NOM) as controls. The expression and distribution of this marker were analyzed in these lesions.

Results: The immunoexpression of PDPN showed a significant increase in the expression of the percentage of positive cells, staining intensity, location of staining in the epithelium, tumor islands, and within the cells, as well as the mean lymphatic micro vessel density between NOMs, OLs, and different grades of OSCCs.

Conclusion: Upregulation of PDPN can be related to the malignant transformation of OLs and biological aggressiveness of OSCCs. The enhanced immunoexpression of PDPN signifies that this immunomarker can have a role in tumor cell differentiation and the neoplastic progression of OSCCs. Increased density of lymphatic vessels suggested an important role of lymphangiogenesis in tumor progression and also as a prognostic factor for lymph nodal metastasis.

## Introduction

Oral squamous cell carcinoma (OSCC) accounts for about 90-95% of all malignancies of the oral cavity [1]. The majority of OSCCs are preceded by visible clinical alterations in the oral mucosa which include oral potentially malignant disorders (OPMDs) such as oral leukoplakia (OL), erythroplakia, and oral submucous fibrosis. The malignant transformation rate of OPMDs to OSCCs is about 6% to 36% [2].

For the past few decades, the mortality rate of OSCC is relatively high with a five-year survival rate of 50% in spite of advancements in diagnosis and treatment modalities [3]. Thus, early diagnosis and appropriate treatment of OPMDs may facilitate the prevention of their malignant transformation into OSCCs [4].

Podoplanin (PDPN) is a mucin-like transmembrane glycoprotein [5]. It is generally expressed in the kidneys, type I alveolar cells of the lung, a few types of neurons, mesothelial cells, osteoblasts, choroid plexuses, glial cells, lymphatic endothelial cells, and various types of fibroblasts [6]. In the oral cavity, PDPN is weakly expressed in the basal layers of the epithelium and strongly expressed in the myoepithelial cells of salivary glands [7]. The physiological role of PDPN includes the normal development of the lymphatic system, lungs, and heart [5]. It is also involved in the morphogenesis of odontoblasts and helps in maintaining the shape of myoepithelial cell processes [7]. It has a role in tumourigenesis, cell motility, tumor invasion, and metastasis [5]. PDPN expression is upregulated in various malignancies such as squamous cell carcinomas of the oral cavity, larynx, skin, lung, esophagus, and cervix, colorectal adenocarcinomas, and testicular germ cell tumors [8].

Carcinogenesis in the oral cavity is a complex mechanism that alters various genes. Immunohistochemical studies allow the identification of these molecular changes as “tumor markers” and contribute to the improved capacity in the diagnosis and evaluation of a prognosis. Thus, there is a need for the identification of biomarkers that detect the changes at a molecular level and predict the malignant transformation of OPMDs such as OLs to OSCCs [9]. Hence, this study was performed to evaluate the role of PDPN as a potential biomarker in predicting the risk of the malignant potential of OLs as well as the tumor progression in OSCCs.

## Materials and methods

This present study was conducted in the Department of Oral Pathology, Microbiology, and Forensic Odontology, Government Dental College and Hospital, Hyderabad, after obtaining the ethical clearance from the Institutional Ethical Committee (Reference number: ECR/300/Inst/AP/2013/RR-16(GDCH-IEC/PG/1922). A total of 75 formalin-fixed, paraffin-embedded tissue sections were selected from the archives of our department. Group I included 15 cases of normal oral mucosa (NOM) as controls that were obtained from vestibular and gingival mucosa during the disimpaction of third molars. Group II included 15 histologically confirmed cases of OLs. Group III comprised 45 cases of OSCC (n=45) with 15 cases each of well-differentiated (WDOSCC) (Group IIIa), moderately differentiated (MDOSCC) (Group IIIb), and poorly differentiated OSCC (PDOSCC) (Group IIIc) that were histologically diagnosed.

The tissue blocks were sectioned at three-micron thickness and placed on 3-amino-propyl-triethoxysilane‑coated slides. Following deparaffinization by heating and treatment with three changes of xylene, the sections were kept in decreasing grades of isopropyl alcohol for rehydration and then immersed in water. The tissue sections were kept in an antigen retrieval solution (Tris-EDTA buffer) and treated at 95°C for three cycles. The antigen-retrieved sections were allowed to cool for 30 minutes and then washed in distilled water followed by rinsing in phosphate-buffered saline. Further, the slides were treated with 3% hydrogen peroxide for 10 minutes to block endogenous peroxidase activity.

The tissue sections were then incubated with a PDPN primary antibody (Pathnsitu Biotechnologies Private Limited, Hyderabad, mouse monoclonal antibody, Clone PM231) for 30 minutes at room temperature, and then the slides were rinsed with a wash buffer. The tissue sections were then treated with a secondary antibody (horseradish peroxidase) at room temperature for 10 minutes. The visualization of the immunohistochemical reaction was performed with freshly prepared substrate chromogen solution (Diaminobenzidine). The sections were then counterstained with Harris hematoxylin and mounted using Dibutyl Phthalate Xylene.

Evaluation of PDPN-positive cells was performed using a compound light microscope at 10x, 20x, and 40x magnifications by two independent observers. The internal positive control was a lymphatic endothelium which had demonstrated a strong PDPN positivity. For each case, five fields of the most representative areas were selected. The immunoreactive score (IRS) given by Remmele and Stegner et al. (Table 1) was calculated for each case [10].

**Table 1:** Immunoreactive scoring system

Percentage of positive cells	x Intensity of staining	= IRS (0-12)	IRS classification
0 = no positive cells	0 = no color reaction	0-1 = negative	0 = negative
1 = <10% of positive cells	1 = mild reaction	2-3 = mild	1 = positive, weak expression
2 = 10-50% of positive cells	2 = moderate reaction	4-8 = moderate	2 = positive, mild expression
3 = 51-80% of positive cells	3 = intense reaction	9-12 = strongly positive	3 = positive, strong expression
4= > 80% positive cells			

The data were evaluated with SPSS Statistics for Windows version 20.0 (IBM Corp. Released 2011. IBM SPSS Statistics for Windows, Version 20.0. Armonk, NY: IBM Corp.). The collected data were analyzed using Pearson’s Chi-square test, one-way ANOVA, and post hoc tests. Confidence intervals were set at 95%, and the results of p<0.05 were interpreted as statistically significant.

## Results

**Figure 1:**
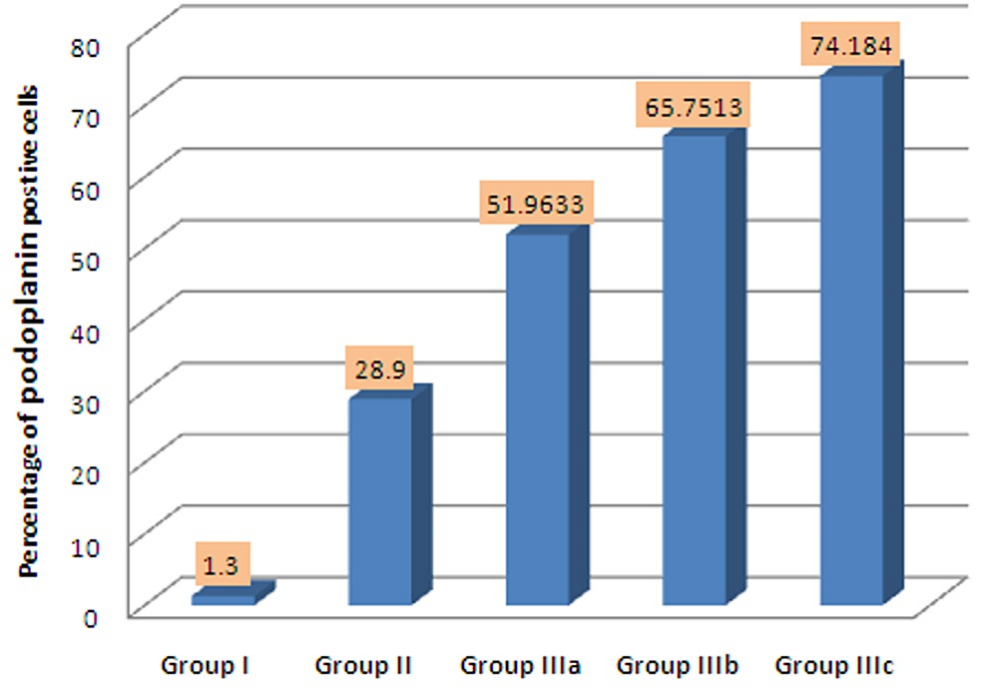
Percentage of PDPN-positive cells between groups I, II, IIIa, IIIb, and IIIc

In our study, the mean percentage of PDPN-positive cells in NOM, OL, WDOSCC, MDOSCC, and PDOSCC were 1.3, 28.9, 51.96, 65.75, and 74.18, respectively. There was a statistically significant increase in the mean percentage of PDPN-positive cells (p=0.003) from NOM to OL to different grades of OSCC (Figure 1).

**Figure 2:**
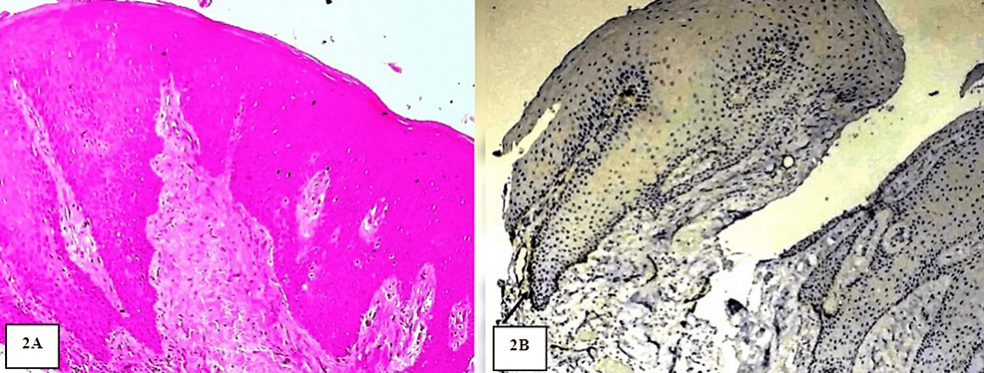
A: Photomicrograph showing features of NOM. Hematoxylin and eosin (H&E) stain (10x). B: Photomicrograph showing focal immunoexpression of PDPN in the NOM. IHC stain (10x)

**Figure 3:**
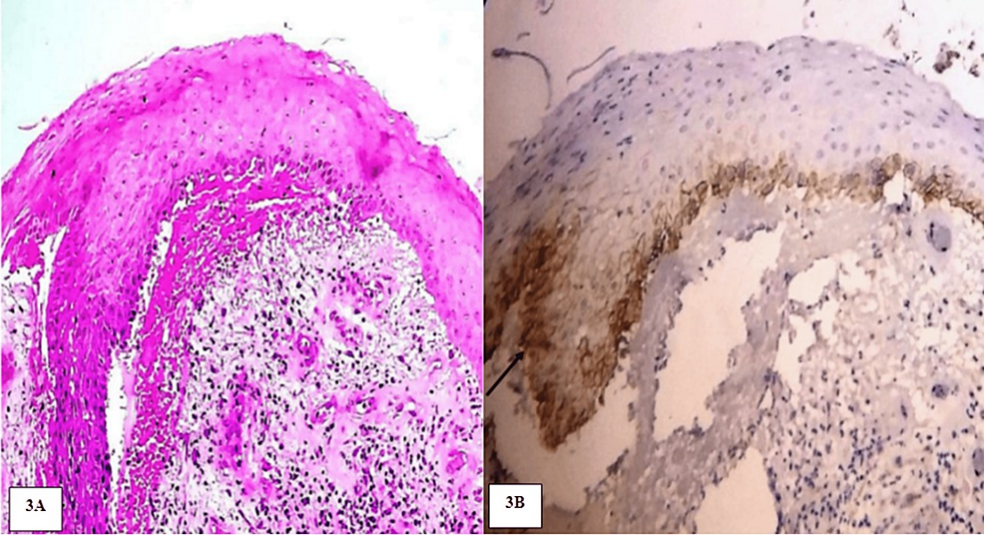
A: Photomicrograph showing OL exhibiting dysplastic features. H&E stain (20x). B: Photomicrograph viewing mild immunoexpression of PDPN in the suprabasal layers of OL. IHC stain (20x)

**Figure 4:**
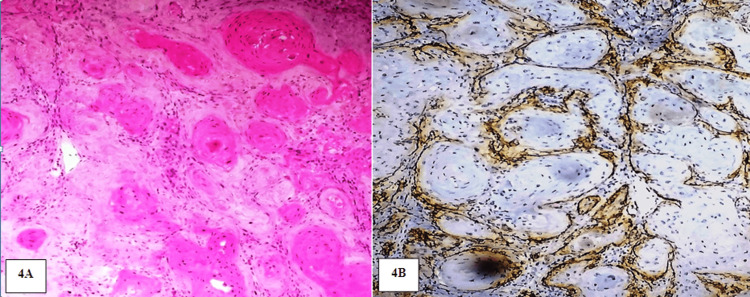
A: Photomicrograph showing WDOSCC. H&E stain (20x). B: photomicrograph showing mild expression of PDPN in the periphery of the tumor islands of WDOSCC. IHC stain (20x)

**Figure 5:**
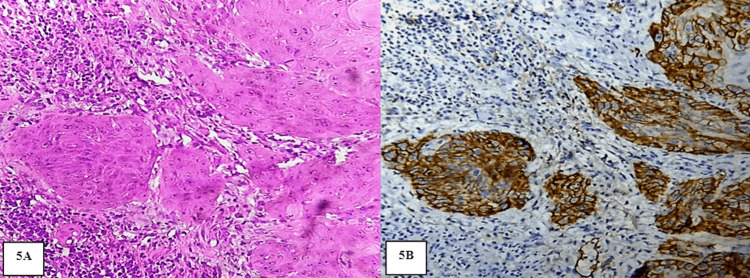
A: Photomicrograph showing MDOSCC. H&E stain (20x). B: Photomicrograph showing moderate expression of PDPN in the periphery and center of the tumor islands in MDOSCC. IHC stain (20x)

**Figure 6:**
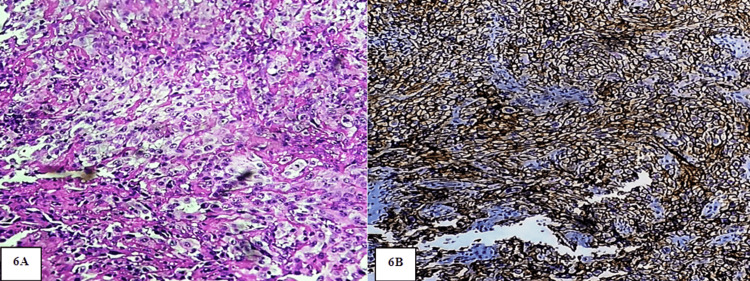
A: photomicrograph showing PDOSCC. H&E stain (20x). B: Photomicrograph showing intense immunoexpression of PDPN in the entire cells of PDOSCC. IHC stain (20x)

In our study, the intensity of PDPN staining was noted. The results showed a statistically significant increase in the intensity of staining from NOM to OL to different grades of OSCC (p=0.042) (Table 2, Figures 2-6).

**Table 2:** Intensity of PDPN staining among groups I, II, IIIa, IIIb, and IIIc

	Mild	Moderate	Intense	Negative	p-value
Group I	4	0	0	11	0.042*
Group II	7	2	0	6	
Group IIIa	8	5	1	1	
Group IIIb	0	10	5	0	
Group IIIc	0	5	10	0	
*Chi-square test, p-value <0.05 was considered statistically significant					

IRS classification was compared between all the groups by Chi‑square test. Our results showed a statistically significant increase in PDPN IRS from NOM to OL to different grades of OSCC (p=0.029) (Table 3).

**Table 3:** IRS classification among groups I, II, IIIa, IIIb, and IIIc

	Negative	Positive weak	Positive mild	Positive strong	p-value
Group I	15	0	0	0	0.029*
Group II	6	6	3	0	
Group IIIa	1	8	5	1	
Group IIIb	0	0	10	5	
Group IIIc	0	0	5	10	
*Chi-square test, p-value <0.05 was considered statistically significant					

The location of PDPN immunoexpression within the epithelium was compared in NOM and OL. In NOM, PDPN expression was detected only in the basal cells of four positive cases. In OL, out of nine positive cases, seven cases were detected in the basal layer and the remaining two cases in the basal and suprabasal layers. There was a statistically significant increase (p=0.028) in PDPN immunoexpression within the epithelium from NOM to OL (Table 4) (Figures 2B, 3B).

 

**Table 4:** Location of PDPN within the epithelium between groups I and II

	Basal layer	Basal and suprabasal layer	Negative	p-value
Group I	4	0	11	0.028*
Group II	7	2	6	
*Chi-square test, p-value <0.05 was considered statistically significant				

**Figure 7:**
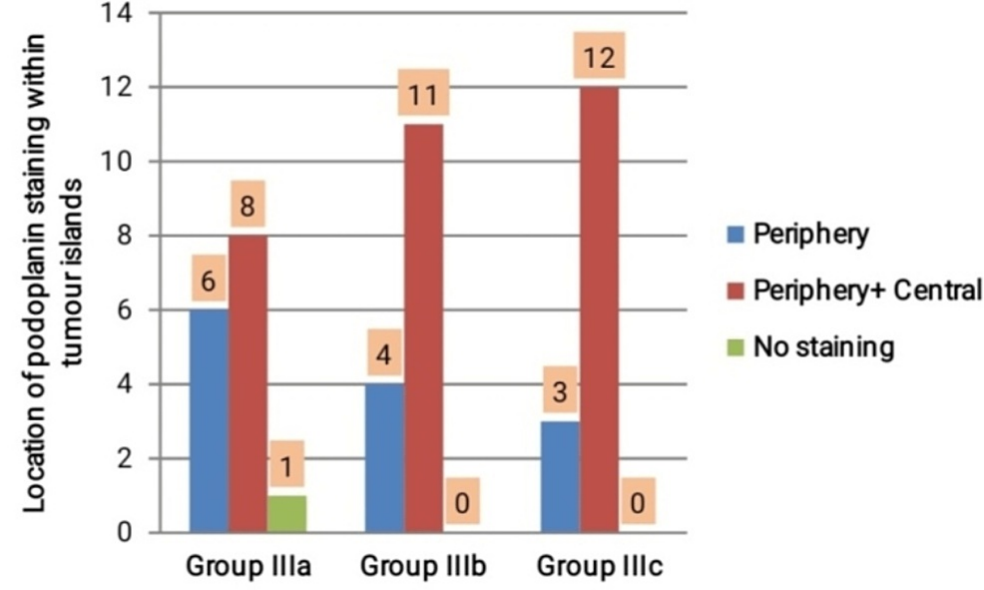
Location of PDPN staining within tumor islands among groups IIIa, IIIb, and IIIc

The location of the PDPN immunoexpression within the invading islands of the tumor cells was compared within all grades of OSCC. In WDOSCC, among 14 positive cases, six cases showed PDPN expression in the tumor cells at the periphery of the islands, and eight cases showed positivity in both central and peripheral cells of the tumor islands. In MDOSCC, 4 out of 15 cases showed positive expression limited to the periphery, whereas the remaining 11 cases showed PDPN expression in the entire tumor islands. Within PDOSCC, the majority of cases (n=12) showed a positive expression in both central and peripheral cells of the tumor islands, and the remaining three cases showed expression in the cells confined to the periphery of the islands. Thus, with increasing in grades of OSCC, the location of PDPN within the tumor islands showed statistically significant results (p=0.003) (Figures 4-7).

**Figure 8:**
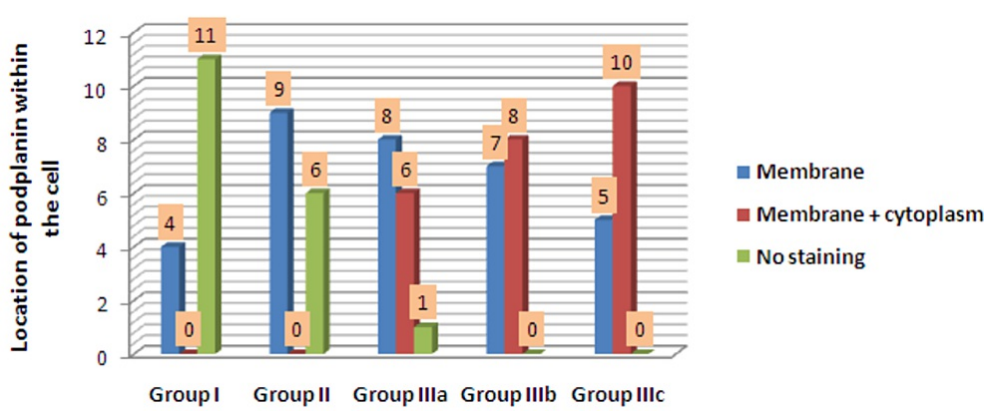
Location of PDPN within the cells among groups I, II, IIIa, IIIb, and IIIc

The location of PDPN immunoexpression within the cells was compared among all the groups. In NOM, PDPN expression within the membrane was noted in all positive cases (4 out of 15 cases). In OL, all the positive cases (n=9) exhibited membranous staining. Within positive cases of WDOSCC (14), eight cases showed membranous staining of PDPN, and six cases showed combined membranous and cytoplasmic staining. Among MDOSCC, eight cases showed combined membrane and cytoplasmic expression and seven cases with membranous staining of PDPN. In PDOSCC, 10 out of 15 showed combined membranous and cytoplasmic expression of PDPN, whereas the remaining five cases showed only membranous staining (Figure 8).

Hence, when there was a shift in histological grades of OSCC from WDOSCC to MDOSCC to PDOSCC, the location of PDPN also exhibited a progressive shift from membranous to a combination of membranous and cytoplasmic expression. There was a statistically significant difference (p=0.041) in the location of PDPN immunoexpression within the cells from NOM to OL to different grades of OSCC.

**Figure 9:**
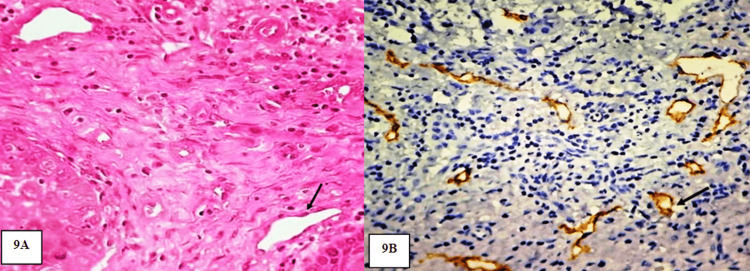
A: Photomicrograph showing lymphatic vessels in PDOSCC. H&E stain (20x). B: Photomicrograph showing PDPN-positive expression in lymphatic vessels of PDOSCC. IHC stain (20x)

In our study, the mean lymphatic micro vessel density (MLVD) was calculated by counting the number of PDPN-positive lymphatic vessels in five highly vascularized fields (under 20x magnification) followed by calculating its mean value (Figure 9).

**Figure 10:**
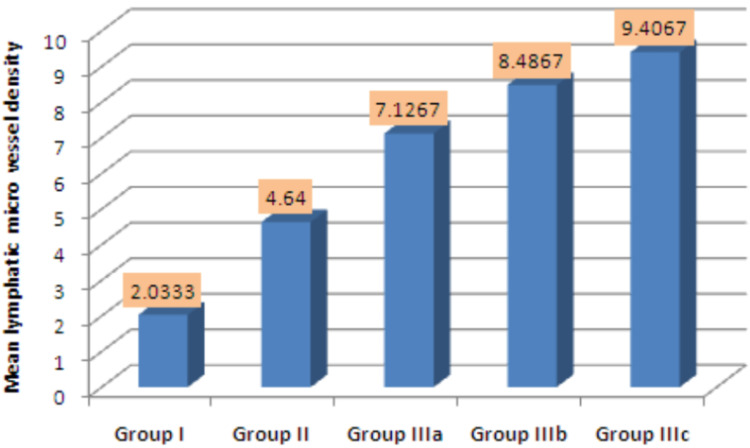
MLVD between groups I, II, IIIa, IIIb, and IIIc

The mean MLVD in NOM, OL, WDOSCC, MDOSCC, and PDOSCC was 2.03, 4.64, 7.13, 8.49, and 9.41, respectively. There was a statistically significant increase in MLVD from NOM to OL to different grades of OSCC (p=0.025) (Figure 10).

The inter-observer agreement is assessed using the Kappa statistical test, and there was an almost perfect agreement between observers 1 and 2 with a Kappa value of 0.966 (Table 5).

**Table 5:** Inter-observer agreement

Observers 1 and 2	Kappa value
	0.966 (almost perfect agreement)
Kappa statistical test	

## Discussion

In the Southeast Asian population, OSCC is considered a foremost distress due to common oral habits such as smoking, alcohol intake, betel quid, areca nut, and tobacco chewing [11,12]. The predisposition of genetic changes like mutations has been found to be another significant risk factor in the occurrence of OSCC [11].

It is striking that most of the OSCCs arise from OPMDs such as OL and erythroplakia. The presence of dysplasia is most significant in predicting the malignant transformation than its clinical appearance in OL and erythroplakia [13]. Hence, the histopathological assessment of epithelial dysplasia is considered the gold standard for assessing the malignant transformation of OPMDs [14]. Previous studies have reported that the malignant transformation rate of OL ranges from 0.13 to 34.0% [15].

Thus, novel molecular markers which help in predicting the malignant potential of OLs and tumor aggressiveness of OSCC are required to be studied extensively in search of potential newer therapies for OSCC and also for its precursors [16].

PDPN is a cell surface protein measuring about 45 Kilodalton which can be induced in basal keratinocytes and dermal fibroblasts during skin remodeling [17]. It is specifically expressed in the endothelium of lymphatic cells but not in the endothelium of blood vessels [5]. PDPN expression is increased in many neoplasms like angiosarcoma, lymphangioma, and squamous cell carcinomas of the oral cavity, skin, esophagus, lung, larynx, and cervix [8].

PDPN expression in early invasive OSCCs is heterogeneous and fragmented, often confined to the invasive tumor front areas [8]. In malignant cells, PDPN is a component of invadopodia, which are actin-rich plasma membrane projections with proteolytic activity. They mediate the degradation of the extracellular matrix components, thereby facilitating the invasion of tumor cells through the epithelial basement membrane [6]. PDPN induces both plasma membrane extension and the actin cytoskeleton rearrangement which favors tumor cell motility [7].

In our study, the mean percentage of PDPN-positive cells showed a statistically significant increase from NOM to OL to different grades of OSCC. Our findings are in accordance with the studies of Logeswari et al. (2014) and Deepa et al. (2017) [18,19]. Thus, PDPN may be regarded as a predictor marker in assessing the malignant transformation of OPMDs like OL and also for the prognosis of OSCC. In contrast, a study by Aiswarya et al. (2019) showed a significant decrease in the percentage of positive cells from WDOSCC to PDOSCC [20].

It was suggested that the induction of PDPN expression in OSCC mediates a pathway mechanism that leads to collective and directional cell migration. In addition, it induces numerous adjustments of intracellular signaling pathways which cause modulation of Rho family GTPase activities, phosphorylation of ezrin, radixin, and moesin proteins, and rearrangement of the actin components, thereby enhancing cell invasion and migration. Therefore, it was concluded that PDPN can have a role in the tumor cell differentiation and neoplastic progression of OSCC [21].

In our study, a statistically significant increase in the intensity of PDPN staining from NOM to OL to different grades of OSCC (p=0.042) was noted. Our results are similar to the findings of Raluca et al. (2015), wherein they noted severe intensity in MDOSCC and PDOSCC compared to WDOSCC. They observed a progressive shift in staining intensity from mild to severe patterns from WDOSCC to PDOSCC [22]. In contrast to our findings, the study results of Aiswarya et al. (2019) showed intense expression of PDPN in WDOSCC, whereas mild intensity was noted in MDOSCC cases [20].

IRS was calculated by multiplying the percentage of PDPN-positive cells and their staining intensity. Our results showed a statistically significant increase in PDPN IRS from NOM to OL to OSCC (p=0.029). With the increase in grades of OSCC from WDOSCC to PDOSCC, there is a tendency toward a positive strong expression of PDPN immunomarker. Our findings are in accordance with the study of Aiswarya et al. (2019), where they observed a statistically significant increase in the mean IRS of PDPN expression from NOM to OL to OSCC [20].

The extent of PDPN immunoexpression in various layers of the epithelium was analyzed in our study which showed a focal positive expression in the basal cells of NOM-positive cases, basal and suprabasal epithelial cells in OLs. Our findings coincided with the study results of Deepa et al. (2017) [19]. Thus, PDPN expression extending to suprabasal layers in some OL cases may represent the upward clonal expansion of stem cells during carcinogenesis. OL cases showing such clonal expansion may imply a significantly higher risk of malignant transformation [23].

In the present study, within different grades of OSCC, the location of PDPN expression was predominantly evident in the entire tumor islands as the grade of OSCC increased. Our observations are similar to the study results of Parhar et al. (2015), wherein they observed a diffuse pattern (both center and peripheral positive cells) of PDPN expression which was prominent in PDOSCC and least in WDOSCC [23]. In contrast to our findings, the study results of Prasad et al. (2015) showed that in WDOSCC, PDPN-positive expression was restricted to the periphery of the tumor nests, whereas the cells in the center of tumor nests showed negativity. Thus, the PDPN expression at the periphery of tumor nests signifies its high proliferative capacity, and central cells suggest terminal differentiation of tumor cells [24].

The location of PDPN expression within the cells was observed and compared. When there was a shift in histological grades of OSCC from WDOSCC to MDOSCC to PDOSCC, the location of PDPN immunoexpression also exhibited a progressive switch from a membrane to a combination of membranous and cytoplasmic expression. Thus, a statistically significant association between the location of PDPN within the cells and different grades of OSCC was noted. In accordance with our findings, the study of Raluca et al. (2015) showed a PDPN expression in both membrane and cytoplasm of tumor cells in different grades of OSCC [22]. In contrast, Laura et al. (2014) noted that the expression of PDPN was limited to membranous staining in cases of poorly differentiated OSCC [25].

Previous studies have suggested that tumor-associated lymphatic vessel formation plays a significant role in tumor progression and metastasis of various malignancies including OSCC [26]. In our study, a statistically significant increase of MLVD from NOM to OL to different grades of OSCC was observed. Within different grades of OSCC, there was a significant increase in MLVD from WDOSCC to PDOSCC. Our results are similar to the study of Aiswarya et al. (2019), in which MLVD was highest in OL and OSCC compared to NOM. Thus, an increase in MLVD in the tumor stroma has been shown to be associated with the metastasis of lymph nodes [20]. In contrast, the study results of Parhar et al. (2015) showed the highest MLVD in MDOSCC cases. Thus, an increase in MLVD in OSCC represents tumoral lymphangiogenesis which may act as an indicator of lymph nodal metastasis in OSCC patients [23].

The majority of OSCCs are diagnosed in the advanced stages. Even after extensive research works using newer diagnostic and prognostic markers, the five-year survival rate in OSCC patients is reported to be very low. This necessitates us to conduct research on new diagnostic and prognostic markers which helps in predicting the malignant potential of OPMDs like OLs as well as to predict the chance of the occurrence of OSCC and its progression, thereby improving the survival rates of patients with OSCC. One such marker is PDPN.

In the present study, the immunoexpression of PDPN was statistically significant in relation to the percentage of positive cells, staining intensity, IRS classification, and location of staining within the epithelium, tumor islands, and cells, as well as MLVD among all the groups.

There are certain limitations in our study like a smaller sample size and the lack of correlation between the risk factors such as smoking, tobacco chewing, and alcohol intake in relation to the occurrence and progression of OL and OSCC.

## Conclusions

This study demonstrated that the upregulation of PDPN expression can be related to the malignant transformation of oral leukoplakias and the biological aggressiveness of OSCCs. From WDOSCC to PDOSCC, there was an increased expression of PDPN with respect to disease severity. The enhanced immunoexpression of PDPN signifies that this immunomarker can have a role in the tumor cell differentiation and neoplastic progression of OSCCs. In OPMDs like OL, showing increased PDPN expression has been found to be related to a high risk of progression to invasive oral cancer, thereby suggesting that it might act as a powerful tumor marker to predict the risk of malignant transformation in OL patients.
